# A Substitute for Copper Amalgam

**Published:** 1894-05

**Authors:** William H. Trueman

**Affiliations:** Philadelphia


### A SUBSTITUTE FOR COPPER AMALGAM.

BY WILLIAM H. TRUEMAN, D.D.S., PHILADELPHIA.

For filling children's teeth, for filling fissures in soft or imperfectly calcified teeth, such as Dr. Perry refers to, page 155, current volume of this journal, and in all cases where from any cause it is desired to use a very plastic amalgam, I have been using for a year or more, with much satisfaction, a heavily silvered alloy, sixty parts of silver to forty parts of tin, using with it sufficient mercury to produce the desired plasticity. If made as recommended by Dr. Flagg, in his excellent work upon "Plastic Fillings," and the alloy cut up with a fine file, or, as I prefer, reduced to delicate mat-surfaced shavings in a turning lathe, a thorough trituration produces a soft plastic mix that with but little pressure is readily and quickly adapted to the cavity by patting with a lock of cotton. It sets very promptly, and makes for these cases a fairly satisfactory filling. It must be borne in mind, however, that an extra amount of mercury favors shrinkage and impairs edge strength, but with heavily silvered alloys to a far less degree than with alloys containing a larger percentage of tin. It takes the place of, and is much to be preferred to copper amalgam. It retains its full plasticity but a few minutes only, and must, therefore, be used quickly.

